# Effects of serial radon spa therapy on pain and peripheral immune status in patients suffering from musculoskeletal disorders– results from a prospective, randomized, placebo-controlled trial

**DOI:** 10.3389/fimmu.2024.1307769

**Published:** 2024-02-06

**Authors:** Anna-Jasmina Donaubauer, Ina Becker, Gerhart Klein, Reinhard E. Voll, Lena Weikl, Martin Klieser, Shakar Barzangi, Jian-Guo Zhou, Rainer Fietkau, Udo S. Gaipl, Benjamin Frey

**Affiliations:** ^1^ Translational Radiobiology, Department of Radiation Oncology, Universitätsklinikum Erlangen, Friedrich-Alexander-Universität Erlangen-Nürnberg (FAU), Erlangen, Germany; ^2^ Department of Radiation Oncology, Universitätsklinikum Erlangen, Friedrich-Alexander-Universität Erlangen-Nürnberg (FAU), Erlangen, Germany; ^3^ Comprehensive Cancer Center Erlangen-EMN, Universitätsklinikum Erlangen, Friedrich-Alexander-Universität Erlangen-Nürnberg (FAU), Erlangen, Germany; ^4^ Association for Radon Spa Research and Medical Practice for Cardiology, Bad Steben, Germany; ^5^ Department of Rheumatology and Clinical Immunology, Medical Centre – University of Freiburg, Faculty of Medicine, University of Freiburg, Freiburg, Germany

**Keywords:** peripheral immune status, radon spa, musculoskeletal disorders, chronic pain, chronic degenerative and inflammatory diseases, randomized placebo-controlled trial

## Abstract

In this randomized, placebo-controlled cross-over trial we aimed to investigate if radon spa therapy exerts more pain relief than exposure to warm water alone. In addition, immunological parameters were assessed in both treatment groups. In the RAD-ON02 trial, 116 patients suffering from musculoskeletal disorders (MSDs) received either serial radon spa or solely warm water baths. Pain intensity was assessed by determination of different pain parameters on a visual analogue scale and by pressure point dolorimetry at baseline and at weeks 4, 12 and 24. The longitudinal immune status of the patients was analyzed by a flow cytometry-based assay from peripheral blood at the time points of pain assessments. There were no side effects attributable to radon exposure observed. However, radon spa was superior to warm water applications at week 4 in terms of pain reduction. Pain and morning stiffness at the time of assessment were significantly reduced after radon spa (p<0.001, p<0.01) but not after warm water baths. The dolorimetry resulted in a significantly higher exerted pressure strength in patients after radon spa (p<0.001), but not after warm water applications. During the long-term follow-up, both treatment modalities reduced pain to a similar degree and pain modulation was not distorted by the participants’ intake of analgesics. No significant changes in the immune status attributable specifically to radon were found, even though the increase in regulatory T cell counts occurs earlier after radon baths than after sole warm water baths and a higher level of significance is reached after radon spa at week 24. Serial radon spa has additive pain-relieving effects. The immunological parameters assessed in our study appear not to be directly linked to the pain reduction caused by radon exposure, at least in MSD patients with predominantly degenerative diseases.

**Clinical trial registration:**

https://www.clinicaltrialsregister.eu/ctr-search/search?query=rad-on02, identifier 2016-002085-31; https://drks.de/search/de/trial, identifier DRKS00016019.

## 1 Introduction

The prevalence of musculoskeletal disorders (MSDs) is constantly rising due to the ageing western population and life style habits favoring obesity and reduced mobility. The term MSD summarizes several disorders of the locomotor apparatus. As MSDs often come along with chronic pain and as 1.7 billion people worldwide suffer from these disorders, they are the leading cause of disability and incapacity for work (1, 2). The therapeutic strategies for MSD comprise predominantly of pharmacological interventions, physical therapy and orthoses as well as surgical interventions in severe cases (1). Often, the disorder cannot be cured and thus requires life-long therapy (3, 4). Hence, there is a huge demand for cost-effective and safe additive therapies, as life-long medication is not only expensive, but also comes along with a marked risk of adverse effects (5–7).

In some countries including Germany, low doses of ionizing radiation (IR) are commonly applied for the treatment of chronic degenerative and inflammatory MSDs. Evidence for the pain-relieving and anti-inflammatory effects of low doses of IR mostly comes from pre-clinical investigations and numerous explorative clinical trials on the use of local serial irradiation with X-Rays on the affected joint. In sum, these investigations found a long-lasting improvement of symptoms, a modulation of the bone metabolism favoring bone formation, as well as an amelioration of inflammatory processes (8–16). However, radiation therapy for MSDs is also commonly applied in health resorts as radon spa therapy using low doses of this radioactive noble gas. Radon can be found in natural fountain water and is absorbed in low doses via the skin by bathing (17–20). Radon spa has a long tradition of application and small doses of about 0.2 to 0.5 mSv are believed to induce pain-relieving and anti-inflammatory effects in patients suffering from different inflammatory and degenerative disorders (21). Nonetheless, the evidence for radon spa therapy is insufficient and mostly comes from the experience of the treating physicians. In line with that, the clinical trials that have been performed so far confirm the analgesic effects of radon spa. Nevertheless, most of these trials lack a placebo control or have major shortcomings in their methodology, especially in study design (17).

The biological mechanisms of action that cause pain relief remain mostly elusive. Nowadays, the amelioration of inflammation, along with a modulation of the bone metabolism, is suggested as a possible mechanism for the pain-relieving effects of radon spa. Evidence for an anti-inflammatory shift of the immune system following therapeutic radon exposure comes from an arthritic mouse model, as well as from several patient studies, including the prospective, observatory RAD-ON01 trial (21–24). In the latter, serum cytokine concentrations, peripheral immune cell counts and their activation state were modulated in patients (21, 25, 26). Besides, the neurosensory system is under discussion as further point of action of radon, as it is able to bind to N-methyl-D-asparte-receptors (NMDARs) just like the noble gas xenon, which is commonly used as anaesthetic in the clinic. Thus, the inhibition of the pathological pain transmission is currently under investigation as mode of action of radon, too (27–30).

Still, prospective trials without placebo control can only provide hints for the beneficial effects of radon spa. Thus, in 2018, we initiated the prospective, double-blind and placebo-controlled RAD-ON02 study. In this trial, the analgesic and immunomodulatory effects of serial radon baths were compared to effects of sole warm water baths. This work presents data on the safety, pain relieve and immunomodulation of serial radon baths.

## 2 Methods and patients

### 2.1 Ethics approval

The RAD-ON02 study (Eudra-CT database no. 2016-002085-31, DRKS database no. DRKS00016019) is a prospective, placebo-controlled, double-blind trial that was conducted according to the German drug law (Arzneimittelgesetz, AMG) at the Bayerisches Staatsbad Bad Steben, Bad Steben, Germany. The trial was performed in accordance with the declaration of Helsinki and was approved by the institutional review board of the Bayerische Landesärztekammer and the Bundesinstitut für Arzneimittel und Medizinprodukte (BfArM). All patients provided written informed consent before enrollment.

### 2.2 Trial design

**Figure 1:**
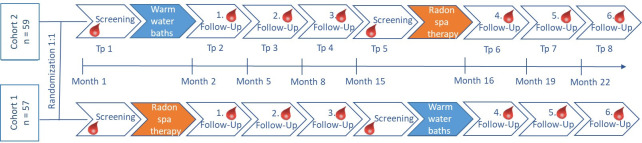
Study scheme of the RAD-ON02 trial. Patients were randomized 1:1 into two clinically comparable cohorts. Assessment of the clinical and biological factors was performed at eight time points (tp) indicated by the blood drops before and after each bath series. For the first half of the study, one cohort received radon baths (orange), while the other cohort received sole warm water baths (blue). Six months after the third follow-up, the patient cohorts switched and the former radon cohort received warm water baths and vice versa.

The patients were randomized in two cohorts and underwent a screening process including the assessment of the initial pain level, the quality of life and the immune status. Then, one cohort received 9 radon baths with a radon concentration of 1200 Bq/L (estimated cumulative dose: 1.3 mGy) and a temperature of 34°C. Each bath lasted for 20 minutes and the treatment was delivered over a period of three weeks. In order to ensure a radon uptake solely via the skin, the bathtubs were covered. The second patient cohort received sole warm water baths without radon. During the three follow-up visits (directly after the bath series as well as 3 and 6 months after the baths) pain, quality of life and immune status were assessed again. One year after the first screening visit, both cohorts underwent a second screening followed by a second bath series. Consequently, the former radon cohort received warm water baths, while the former placebo cohort received radon baths, respectively. The outcomes were assessed again as described before. During the whole study period, the patients were supposed to document their medication intake on a weekly basis. The study scheme is depicted in **Figure 1**.

### 2.3 Patient inclusion and exclusion criteria

Patients >40 years of age suffering from MSDs of the spine and joints (pain duration ≥ 1 year, pain intensity ≥ 4 on a visual analogue scale (VAS), ranging from 0-10) were eligible for enrollment. All patients were supposed to be located around Bad Steben in order to minimize environmental differences and to rule out placebo effects from spa-holiday benefits. Patients suffering from uncontrolled arterial hypertension, congestive heart disease or acute inflammatory diseases were not eligible. A preceding radon spa or oncological radiation therapy were exclusion criteria.

### 2.4 Randomization and blinding

Patients were randomized 1:1 by an independent statistician under consideration of the main clinical characteristics, such as the age and gender, to result in clinically comparable cohorts. In order to avoid accidental unblinding by characteristic smell of the radon bath water (the thermal water comes from a fountain that passes through several layers of soil and rock which leads to the adsorption of compounds that cause a characteristic smell), a strong-smelling bath additive containing herb extracts was used in all baths. The patients, the treating physicians, as well as the involved scientists were blinded.

### 2.5 Endpoints

The primary endpoint was the determination of the detailed, longitudinal immune status of the patients in order to discriminate the immunomodulatory effects of radon spa from sole warm water baths by a flow cytometry-based assay and by the quantification of inflammatory mediators in serum. Further, the determination of the pain reduction following the baths was a major goal and assessed by questionnaires (VAS), pressure point dolorimetry, and medication diaries.

### 2.6 Assessment of pain

The pain was assessed by detailed questionnaires allowing the patients to score different pain parameters on a VAS during the visits. To receive more objective data, pressure point dolorimetry was performed additionally and the data for the most painful pressure point is presented here. The applicable pressure to induce pain was determined for 8 pressure points according to general practice (31). Additionally, patients documented their drug intake (including the dosage) on a weekly basis to quantify the intake of pain medication.

### 2.7 Immunophenotyping

Longitudinal immunophenotyping was performed to determine the detailed immune status of the patients within 6 hours after the blood withdrawal by multicolor flow cytometry. The detailed procedure is described in our previously published protocols (32–34). The gating strategy for the determination of the immune cell types and their respective activation status can be found elsewhere. A Gallios flow cytometer (Beckman Coulter) in the standard filter configuration was used for data acquisition. The data analysis was performed using the Kaluza Flow Analysis Software (Beckman Coulter).

### 2.8 Statistical analyses

Microsoft Excel 2016 was used for data collection and management. The statistical analyses, including the descriptive statistics (Mean, Median, SEM, 95% CI), were performed in Microsoft Excel 2016 and R (version 4.2.1).

The sample size was calculated based on a paired t-test, as suggested by Dupont and Plummer (35). A power of 0.8 and a level of significance of ≤0.05 was considered. To achieve a higher number of cases for each treatment modality, the data sets of both arms were pooled for the respective treatment. To ensure that the six month “wash-out phase” was sufficient and no immune- or pain-related effects were carried-over in the second half of the study, a t-test was applied. No carry-over effect into the second half of the study was confirmed for any parameter.

For the inductive statistical analyses, the two-sided paired t-test was applied to test, if there is a significant difference in the measured factors in the same individual between the different time points. For several parameters the descriptive statistic displayed a strong deviation from the normal distribution, thus the non-parametric Wilcoxon-signed rank test was applied for those parameters. In the figure legends the utilized test is indicated.

The graphical illustration was performed with GraphPad Prism 9.

## 3 Results

### 3.1 Patient population

**Figure 2:**
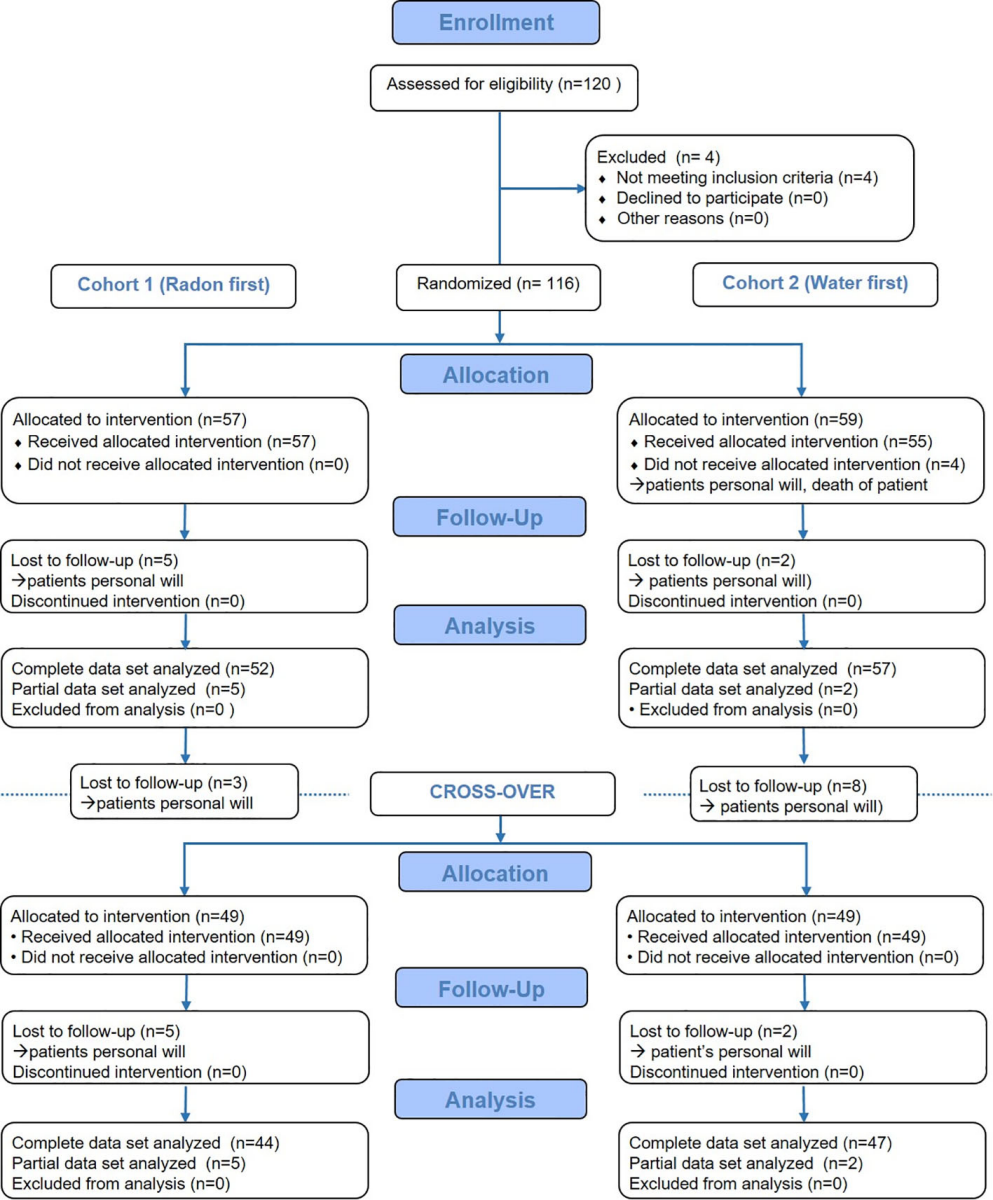
Flow diagram on the course of the RAD-ON02 trial. The diagram depicts the course of the study and displays the number of patients that were assessed for eligibility, were allocated to the intervention, were followed-up and finally analysed. The patient numbers are displayed for both study arms (cohort 1 and cohort 2) and for the interventions before and after the cross-over separately.

In the RAD-ON02 trial, 116 patients were enrolled in November 2018 and followed-up until July 2020. The flow diagram in **Figure 2** describes in the detail the number of patients that were assessed, enrolled and analysed, as well as the number of the drop-outs. The drop-out rate over the total study time was low. There was no indication for a link between the drop-outs and the applied treatment (radon or sole warm water spa), because there were comparable dropout rates after both treatment modalities. Mostly, the reason for the drop-out was the patient’s personal will.

The respective clinical characteristics of the participants are described in **Table 1** for the entire patient collective, as both cohorts were pooled for the here presented analyses. Further, an additional table [see additional **Table 1**] shows the patients’ characteristics stratified by cohorts. The mean age at the start of the study was 59 years and around two thirds (63%) of the cohort were female. More than 60% of the participants were overweight (43.1%) or obese (21.6%), compatible with obesity being a risk factor for MSDs. Concerning the relevant disorders, the patients were grouped on whether only the big joints (20.7%), only the spine (9.5%) or multiple structures were affected (69.8%).

**Table 1:** Demographic and clinical patient characteristics.

Factor	Category	n
Total number		116
Age at start (years)	Mean	59
	Range	39-75
Sex assigned at birth	Male	43 (37%)
	Female	73 (63%)
BMI	Normal (< 25)	28 (24.1%)
	Overweight (25–30)	50 (43.1%)
	Obese (> 30)	25 (21.6%)
	N/A	13 (11.2%)
Indication	Big Joints	24 (20.7%)
	Spine	11 (9.5%)
	Multiple Indications	81 (69.8%)

### 3.2 Assessment of pain and morning stiffness

**Figure 3:**
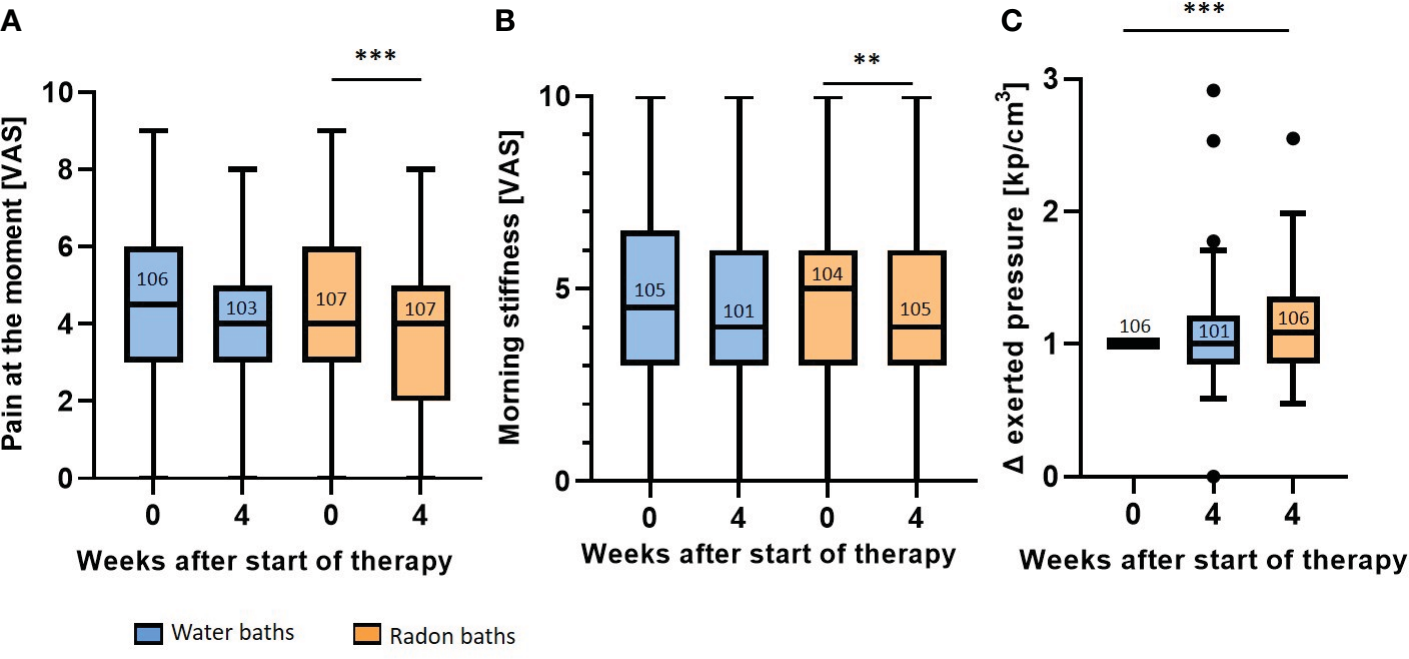
Radon spa induces a stronger pain reduction than sole warm water applications. The pain at the moment **(A)** and the morning stiffness **(B)** were determined on a VAS. The pressure strength that is needed to induce pain was described in kp/cm3 **(C)**. The pressure applied after each bath series was normalized to the initial score. All parameters were assessed before therapy (week 0) and one week after the end of the treatment (week 4). The lines in the bars indicate the median score and the numbers depict the amount of independent data sets available. A paired t-test was applied for the comparisons in **(A, B)**. The Wilcoxon test was applied in **(C)**. The whisker in the boxplots were calculated by the Tukey method. Data points that were laying outside of these borders were plotted as black dots. **p<0.01; ***p <0.001.

The RAD-ON02 trial proved that radon spa is superior to sole warm water applications directly after the bath series in patients suffering from MSDs. The results of the pain assessment before and directly after the baths are presented in **Figure 3**.

For the parameter *pain at the moment* (**Figure 3A**), patients receiving sole warm water had a median score of 4.5 (mean 4.4, 95% CI 4.8-4.0) on the VAS before treatment, that declined to a score of 4 (mean 4.0, 95% CI 4.5-3.7) directly after the baths. This reduction in the VAS pain after warm water applications was not statistically significant. In contrast, after radon spa, a significant pain reduction (p<0.001) was observed. Even though the initial median scores before as well as directly after radon baths was 4 on VAS, the mean VAS score decreased from 4.4 (95% CI 4.8-4.0) to a mean VAS score of 3.8 (95% CI 4.2-3.4) in week 4. Further, the evaluation of the *morning stiffness* also showed that radon spa was superior to placebo (**Figure 3B**). After water baths, the median score declined from 4.5 (mean 4.6, 95% CI 5.1-4.2) to 4 (mean 4.4, 95% CI 4.9-4.0) on the VAS. Still, the reduction was not statistically significant. Radon spa however, led to a significantly lowered *morning stiffness* of the joints (p<0.01). The median score on the VAS decreased from 5 (mean 4.8, 95% CI 5.3-4.4) to 4 (mean 4.4, 95% CI 4.8-4.0). For a more objective pain assessment, pressure point dolorimetry was performed (**Figure 3C**). In line with the previous results (see **Figures 3A, B**), a significant pain reduction was only observed after radon spa (p<0.001), but not after placebo treatment. After the radon application, the applied median pressure was increased 1.08 times (mean 1.13, 95%CI 1.2-1.07) in relation to the initial score, while water baths did not lead to an increased pressure strength (week 4: median 1.0, mean 1.06, 95% CI 1.13-1.0).

### 3.3 Immune modulation

**Figure 4:**
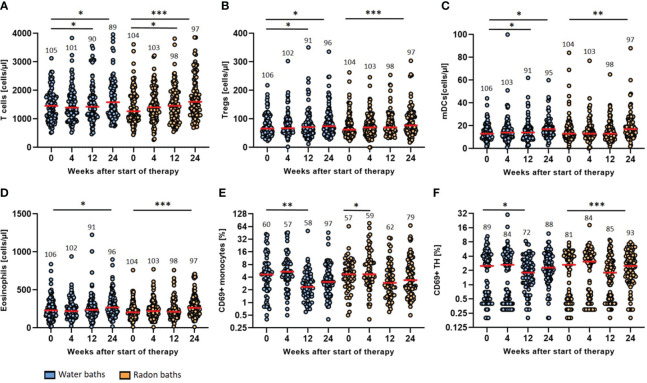
Radon spa therapy leads to longitudinal immune modulation that is related to general spa effects. The absolute cell counts (cells/µl) of T cells **(A)**, Tregs **(B)**, mDCs **(C)** and eosinophils **(D)** as well as the activation state (% positive cells) of monocytes **(E)** and TH cells **(F)** determined by surface expression of CD69 were quantified by detailed immunophenotyping. All parameters were assessed before the baths (week 0) as well as in week 4, 12 and 24. The red lines indicate the median counts/percentages and the numbers show the amount of independent data sets available. A paired t-test was applied to all data sets. *p <0.5; **p<0.01; ***p <0.001.

As immunomodulation is discussed as possible mode of action of radon spa therapy, longitudinal immunophenotyping was performed to detected changes in the peripheral immune system that might be attributable to radon spa. However, no striking differences in the immune parameters analyzed were found after warm water compared to radon baths. For all immunological parameters presented here, we found significant modulations after radon spa, but also after sole water baths (**Figure 4**), even though with small tendencies of different dynamics. As hints on immunomodulation following radon spa came from our previous, observatory trial (21), representative parameters that were modulated in the preceding trial are presented here.

The general T cell count increased significantly three and six months after radon spa (p<0.05; p<0.001) and warm water spa (p<0.05; p<0.05) (**Figure 4A**). The median T cell count increased from 1458 cells/µl (mean 1477, 95% CI 1580-1374) at baseline to 1586 cells/µl (mean 2132, 95% CI 2729-1535) six months after warm water baths and from a median count of 1267 cells/µl (mean 1427, 95% CI 2729-1535) to 1603 cells/µl (mean 1771, 95% CI 1922-1621) six months after radon spa, respectively. A significant rise of the count of regulatory T cells (Tregs) was detected as well after both treatment modalities (**Figure 4B**). After the application of warm water, a significant increase of the cell counts was detected three and six months after the baths (p<0.05; p<0.05) from a median initial count of 66 cells/µl (mean 77, 95% CI 84-69) to a final median count of 75 cells/µl (mean 114, 95% CI 151-77). A significant increase of Tregs was noticed as well six months after the application of radon spa (p<0.001) from a median count of 62 cells/µl (mean 75, 95% CI 83-68) to 77 cells/µl (mean 90, 95% CI 100-80). Moreover, the myeloid dendritic cells (mDCs) showed higher counts in peripheral blood after the application of either treatment (**Figure 4C**). A significant increase after water application was found three and six months later (p<0.05; p<0.05) from a median count of 13 cells/µl (mean 14, 95% CI 16-13) initially to 17 cells/µl (mean 29, 95% CI 41-16) six months later. For radon spa, a significant rise of the cell counts was only noticeable six months after the baths (p<0.01) from a median count of 13 cells/µl (mean 16, 95% CI 18-14) to 17 mDCs/µl (mean 20, 95% CI 22-17) at the end of the trial. In line with the previous results, the count of the eosinophilic granulocytes was rising as well six months after both bath series (**Figure 4D**). The eosinophils increased from a median initial count of 231 cells/µl (mean 283, 95% CI 357-209) to 270 cells/µl (mean 442, 95% CI 629-255) (p<0.05) six months after the placebo treatment and from a median count of 205 cells/µl (mean 227, 95% CI 248-215) to a final count of 264 cells/µl (mean 290, 95% CI 320-261) (p<0.001) after radon spa, respectively.

The evaluation of the activation status of the immune cell populations showed a decrease in the expression of the majority of activation markers on the analyzed immune cell populations, especially three and six months after the baths (**Figure 4**). CD69 was modulated on different immune cells in particular. On monocytes, CD69 showed a reduced expression three months after the warm water baths (p<0.01), from a median percentage of 4.7% (mean 7.6, 95% CI 9.9-5.2) CD69+ monocytes to 2.4% (mean 3.9, 95% CI 5.6-2.2). A significant decrease at week 4 after treatment was only observed for the radon baths (p<0.05). However, the median count of CD69+ monocytes did not change after radon spa (median 4.7, mean 10.5, 95% CI 14.0-6.9). Even though not significant, a further, persisting decrease of CD69+ monocytes was observable after both treatment modalities. In agreement, the percentage of CD69+ T Helper (TH) cells decreased as well after both interventions. For the warm water baths, a significant decrease was recorded three months after the baths (p<0.05) from a median percentage of 2.5% (mean 2.6, 95% CI 3.1-2.1) CD69+ TH cells to 1.8% (mean 2.3, 95% CI 2.7-1.9) positive TH cells. Six months after radon spa, a significant decrease (p<0.001) of CD69+ TH cells was observed from an initial median percentage of 2.7 (mean 2.6, 95% CI 3.0-2.1) to a final percentage of 2.5 (mean 3.0, 95% CI 3.3-2.6), giving hint for slightly different dynamics of modulations induced by radon spa compared to warm water baths.

### 3.4 Long-term outcome

**Figure 5:**
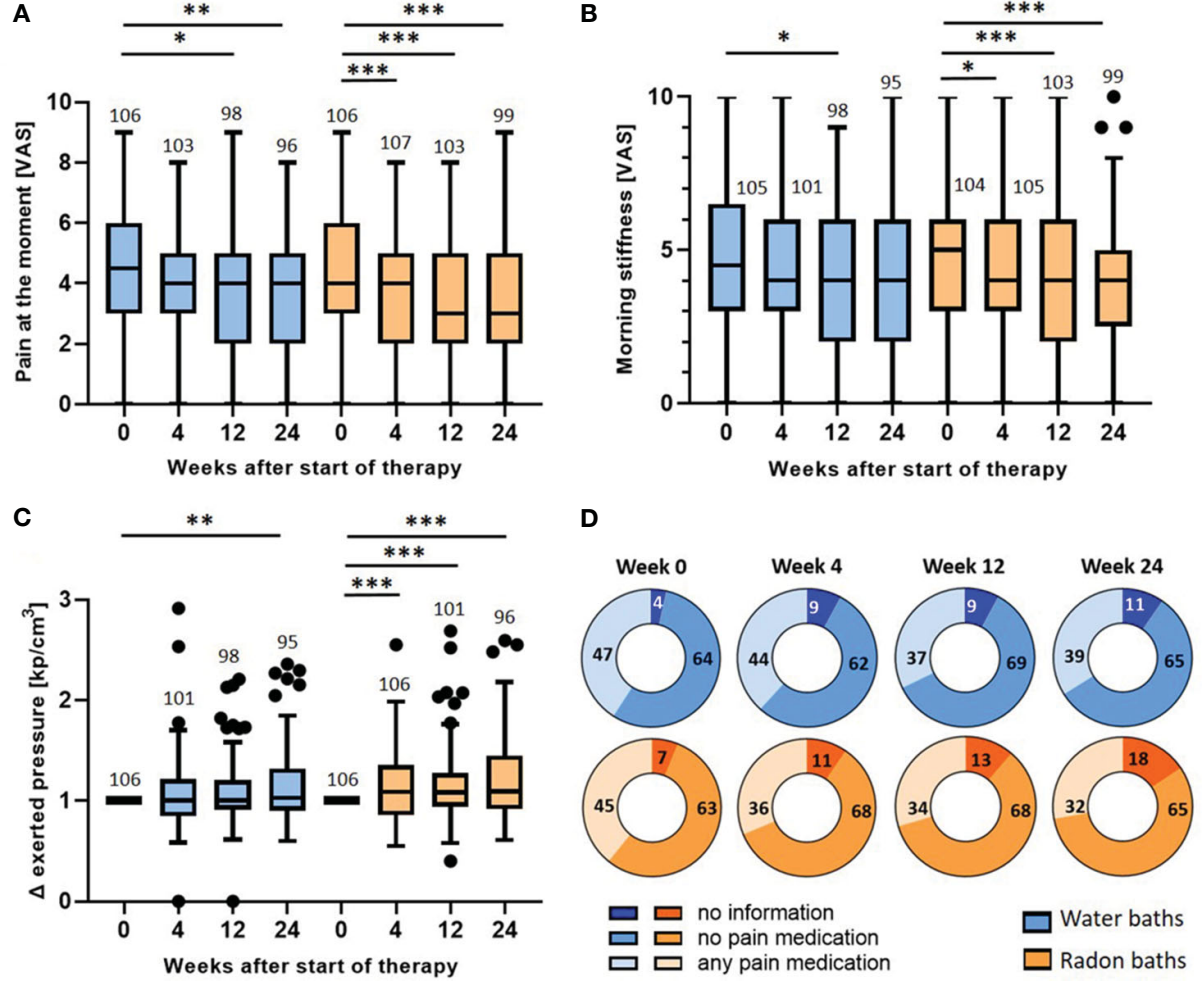
Longitudinal evaluation of pain parameters: wash-out of the pain relief and evaluation of medication intake. The pain at the moment **(A)**, the morning stiffness **(B)** and the pressure strength needed to induce pain **(C)** were determined longitudinally before the baths (week 0) and 4, 12 and 24 weeks after the bath series. The parameters in **(A, B)** were determined on a VAS. In **(C)** the exerted pressure was assessed by dolorimetry (kp/cm3). The pressure strength after the bath series (week 4, 12 & 24) was normalized to the initial pressure strength (week 0). The red lines indicate the median counts/percentages and the numbers show the amount of independent data sets available. The medication intake **(D)** was quantified in week 0, 4, 12 and 24 and depicted as absolute number of patients that took analgesics, patients that did not take medication and patients that gave no information. The paired t-test was applied in **(A, B)**. In **(C)**, the Wilcoxon test was applied. The whisker in the boxplots were calculated by the Tukey method. Data points that were laying outside of these borders were plotted as black dots. *p <0.5; **p<0.01; ***p <0.001.

As the immunological modulations were mostly persistent until the end of the trial, the pain parameters were also assessed longitudinally (**Figure 5**).

As presented in **Figure 3**, the parameters were only significantly altered directly after radon spa but not after warm water. For the parameter *pain at the moment* (**Figure 5A**), the evaluation three and six months after the bath series showed a further pain reduction after radon spa that is significantly lower than the initial pain level (week 0) (p<0.001; p<0.001) from a median initial score of 4 (mean 4.4, 95% CI 4.8-4.0) to a final score of 3 (mean 3.6, 95% CI 4.0-3.2) on VAS. However, a significant, and comparable pain reduction from the initial pain score in week 0 was also observed three and six months after the water baths (p<0.05; p<0.01) from an initial median score of 4.5 (mean 4.4, 95% CI 4.8-4.0) on the VAS to a final score of 4 (mean 3.7, 95% CI 4.1-3.3) six months later. Likewise, a further pain reduction was also detected for the parameter *morning stiffness* (**Figure 5B**). After radon spa, the *morning stiffness* decreased significantly three and six months post therapy (p<0.001; p<0.001) from an initial median score of 5 (mean 4.8, 95% CI 5.3-4.4) on VAS to a final score of 4 (mean 4.0, 95% CI 4.4-3.5). After warm water baths however, a significant reduction of the pain was only observed three months after baths (p<0.05) from initially 4.5 (mean 4.6, 95% CI 5.1-4.1) on VAS to 4 (mean 4.1, 95% CI 4.5-3.6). The longitudinal evaluation of the dolorimetry confirmed the preceding results (**Figure 5C**). After radon spa, a significant increase of the applied pressure was observed three and six months after the baths (p<0.001; p<0.001) as well. Finally, the exerted median pressure increased 1.09 times (mean 1.2, 95% CI 1.29-1.12) in comparison to the initially applied pressure. After warm water applications, a significant increase of the exerted pressure was only observed six months after baseline (p<0.01) with the median applied pressure increasing 1.03 times (mean 1.15, 95% CI 1.23-1.07).

To ensure that the data on the pain modulation is not distorted by the participants’ intake of analgesics, the pain medications were recorded and quantified throughout the study (**Figure 5D**). Of note, no significant change in the absolute number of patients that took pain medication was observed after radon spa or warm water baths, neither 4, nor 12 or 24 weeks after the baths. In fact, the data even shows a tendency towards a lowered medication intake 4 weeks after radon spa, whereas numerically more patients took analgesics after the water baths.

### 3.5 Safety and side effects

In sum, 13 patients receiving radon spa and 9 patients receiving sole warm water reported therapy-related adverse effects. After radon spa, two adverse events were reported, which were vaginitis and the exacerbation of a pre-existing neurodermitis. The latter one was not attributable to the radon spa, but due to an allergic reaction against the bath additives, according to the attending physician. All other reported side effects required no intervention. The events appeared only once or twice in each group after one of both interventions. Thus, most likely none of the events was attributable to radon alone. All events are listed in detail in **Table 2**. Importantly, all events appeared in close timely proximity to the bath series and no side effects were reported three and six months after the baths.

**Table 2:** Adverse events & side effects.

Factor	Category		n
		Radon cohort	Water cohort
Total number		116	116
Adverse events	Vaginitis	1 (0.9%)	0 (0%)
	Exacerbation of neurodermitis	1 (0.9%)	0 (0%)
Side effects	Hypertension	1 (0.9%)	1 (0.9%)
	Vascular complaints	1 (0.9%)	0 (0%)
	Tingling sensations (legs)	1 (0.9%)	0 (0%)
	Pruritus & dry skin	1 (0.9%)	2 (1.8%)
	Nausea	1 (0.9%)	0 (0%)
	Headache	1 (0.9%)	0 (0%)
	Sweating & elevated body temperature	2 (1.8%)	1 (0.9%)
	Insomnia	1 (0.9%)	0 (0%)
	Short term increase in joint pain	1 (0.9%)	1 (0.9%)
	Abdominal pain Leg edema	1 (0.9%) 0 (0%)	0 (0%) 1 (0.9%)
	Fatigue	0 (0%)	1 (0.9%)
	Thoraric pain	0 (0%)	1 (0.9%)
	Cold extremities	0 (0%)	1 (0.9%)

## 4 Discussion

The RAD-ON02 trial demonstrated that serial radon spa induces a long-lasting pain relief in patients suffering from MSDs. The subjective scoring on the VAS, but also the dolorimetry demonstrated that radon spa was superior to warm water at week 4. During the long-term follow-up, further significant pain reductions were observed 12 and 24 weeks after radon spa as well as after water applications, however, without a significant difference between both interventional groups.

The long-term pain reduction presented in this study is in line with previous trials. For instance, the observatory RAD-ON01 trial found a comparable pain reduction that lasted at least 30 weeks after radon spa therapy in MSD patients (21). A non-blinded comparison of radon spa and sauna therapy for ankylosing spondylitis stated a stronger pain reduction after radon spa that lasted for at least three months (36). Still, these trials lack a placebo-control that is indispensable to discriminate radon-specific effects from the effects of warm water. Thus, Franke et al. performed randomized trials on radon spa that stated an advantageous effect of radon over water applications. However, they found that both interventions were initially comparable but the effect lasted longer after radon spa (17, 19, 37, 38). In the RAD-ON02 trial however, we found radon spa to be superior to warm water baths only during the short-term follow-up (week 4). These divergent results might be due to differences in the study design and a rather inflammatory than degenerative background of the patient collective in the trials of Franke et al. Nevertheless, the findings of the RAD-ON02 study and the other mentioned trials demonstrate that radon spa induces analgesic effects that can be separated from the pain-relieving effects that are induced by warm water baths.

Further evidence for the therapeutic benefit of radon spa comes from the quantification of the medication intake. The reported pain reduction was not biased by an increased or decreased consumption of pain medication, as neither after radon spa nor after warm water a significant change in the intake of pain medication was observed. We even noticed the tendency towards a lowered need for pain medication after radon spa that was not found after sole water baths.

Even though we found radon spa to be superior to warm water baths initially, sole warm water baths also induced analgesic effects that were found to be significant in week 12 and 24. In fact, bathing itself is known to reduce pain and can be applied as supportive therapy for MSDs. Trials on different MSDs, such as fibromyalgia or osteoarthritis confirmed a pain relief after serial baths that can be discriminated from patients not receiving any therapy (39, 40).

In sum, our results demonstrated a pain-relieving effect of radon spa. Thereby, especially the short-term pain relief can be predominantly attributed to the very low doses of radon, whereas in the long term the warm water baths effects may dominate, at least in degenerative MSDs.

A second major endpoint of the RAD-ON02 trial was the evaluation of the longitudinal immune status after radon spa, as immunomodulatory effects are under discussion as a mode of action of radon spa. For therapy with X-rays, the modulation of the immune system and the amelioration of inflammation has already been confirmed in preclinical models and in clinical investigations (8, 9, 11, 41, 42). In agreement, longitudinal systemic immune modulations in patients suffering from MSDs have also been detected in the observatory RAD-ON01 trial (21, 26). As also degenerative musculoskeletal disorders frequently come along with low level chronic inflammation that propagates tissue destruction, pain and stiffness, our hypothesis was that immune modulation might be responsible for the pain relief after radon spa.

While screening numerous immune cells parameters for their modulation by radon spa, we put a special emphasis on those parameters that were already shown to be modulated in the preceding RAD-ON01 trial (21). For many parameters that were modulated in the latter trial, we did also find significant alterations in the blood of patients of the RAD-ON02 study. Noteworthy, those modulations occurred not only after radon spa, but also after the application of warm water, even though in slightly different dynamics. Thus, with respect to the data analysed here, we could not identify an immunomodulatory effect attributable directly to radon spa. The lowered expression of the activation marker CD69, as well as the increased counts of different innate and adaptive immune cell populations were equally pronounced after radon and warm water baths. However, one can notice that the increase in regulatory T cell counts occurs earlier after radon baths than after sole warm water applications and a higher level of significance is reached for the modulations after radon spa (see **Figure 4B**). This finding is in line with the observations of Eckert et al, who analyzed the regulatory T cell to Th17 ratio in a subgroup of patients of the RAD-ON02 study via intracellular FACS staining, and found a significant increase of regulatory T cells only after radon spa (43). Some studies state that spa applications in general impact on the immune system, for example by the modulation of cytokines, adipokines and other serum proteins, which is in line with the immune modulations that have been observed after radon and warm water applications. These modulations are most likely due to a hyperthermia effect of the warm water (44–46). Even though our results implicate that immunomodulation might not be the primary mode of action of the radon-induced pain relief in MSD patients, in a murine model on acute inflammatory arthritis, radon application has led to a significant increase of circulating B cells and IL-5 along with significantly improved disease progression that was not observed in mock-treated mice (25). Although these modulations were not found in this real-world patient cohort of the RAD-ON02 study (**Supplementary Figure 1**), radon-induced immunomodulation might still be relevant in more inflammatory-driven disease settings.

As we saw that radon spa was superior to sole warm water in terms of initial pain relief, further modes of action might play a role that are responsible for the radon spa specific analgesic effect in the here presented patient cohort. As radon shares many physicals characteristics with the noble gas xenon that is a potent anaesthetic, it might as well impact on the neurosensory system. In fact, radon is also able to bind NMDA receptors, thus, the inhibition of pain transmission might be a further point of action of radon (27, 30, 47). Since chronic pain and a pathological pain transmission is a key feature in chronic MSDs, this biological mode of action is likely to be involved in the radon-induced pain relief.

We found that radon spa was superior to sole warm water baths in terms of pain relief after the bath series. Nonetheless, in patients suffering from MSDs, this pain relief is not induced by an amelioration of inflammatory processes. Thus, further modes of action are likely to play a role, such as the inhibition of pain transmission. Future trials should not only focus on more inflammation-driven diseases such as axial spondylarthritides to prove the immunomodulatory effect of radon, but also put further emphasis on other suggested modes of action of radon spa, such as the impact on the neurosensory system. In sum, the prospective and placebo-controlled RAD-ON02 trial increases the evidence for radon spa therapy and provides the foundation for future randomized trials and mechanistic analyses.

## Data availability statement

The raw data supporting the conclusions of this article will be made available by the authors, without undue reservation.

## Ethics statement

The studies involving humans were approved by Institutional review board of the Bayerische Landesärztekammer and the Bundesinstitut für Arzneimittel und Medizinprodukte (BfArM). The studies were conducted in accordance with the local legislation and institutional requirements. The participants provided their written informed consent to participate in this study.

## Author contributions

A-JD: Conceptualization, Formal analysis, Investigation, Methodology, Software, Validation, Visualization, Writing – original draft. IB: Investigation, Methodology, Software, Writing – review & editing. GK: Conceptualization, Project administration, Writing – review & editing. RV: Writing – review & editing. LW: Formal analysis, Investigation, Validation, Writing – review & editing. MK: Formal analysis, Writing – review & editing. SB: Formal analysis, Writing – review & editing. J-GZ: Data curation, Formal analysis, Methodology, Software, Validation, Writing – review & editing. RF: Conceptualization, Funding acquisition, Resources, Writing – review & editing. UG: Conceptualization, Resources, Supervision, Validation, Visualization, Writing – review & editing. BF: Conceptualization, Funding acquisition, Methodology, Project administration, Resources, Supervision, Validation, Visualization, Writing – review & editing.
