# Supplementary material for: Effects of serial radon spa therapy on pain and peripheral immune status in patients suffering from musculoskeletal disorders– results from a prospective, randomized, placebo-controlled trial
Source: Front Immunol. 2024 Feb 6;15:1307769. doi: 10.3389/fimmu.2024.1307769 (PMC10876773; doi:10.3389/fimmu.2024.1307769)
Supplement: Supplementary file 1 [file DataSheet_1.docx]

Supplementary Material

# Supplementary Tables

**Supplementary Table 1. Demographic and clinical patient characteristics stratified by cohorts**

| **Factor** | **Category** | **n** | |
| --- | --- | --- | --- |
|  |  | Cohort 1 | Cohort 2 |
| Total number |  | 57 | 59 |
| Age at start | Mean | 59 | 59 |
|  | Range | 40-73 | 39-75 |
| Gender | Male | 23 (40·4%) | 20 (33·9%) |
|  | Female | 34 (59·6%) | 39 (66·1%) |
| BMI | Normal (< 25) | 9 (15·8%) | 19 (32·2%) |
|  | Overweight (25-30) | 32 (56·1%) | 18 (30·5%) |
|  | Obese (> 30) | 11 (19·3%) | 14 (23·7%) |
|  | N/A | 5 (8·8%) | 8 (13·6%) |
| Indication | Big Joints | 11 (19·3%) | 14 (23·7%) |
|  | Spine | 6 (10·5%) | 5 (8·5%) |
|  | Multiple Indications | 40 (70·2%) | 40 (67·8%) |

# Supplementary Figures


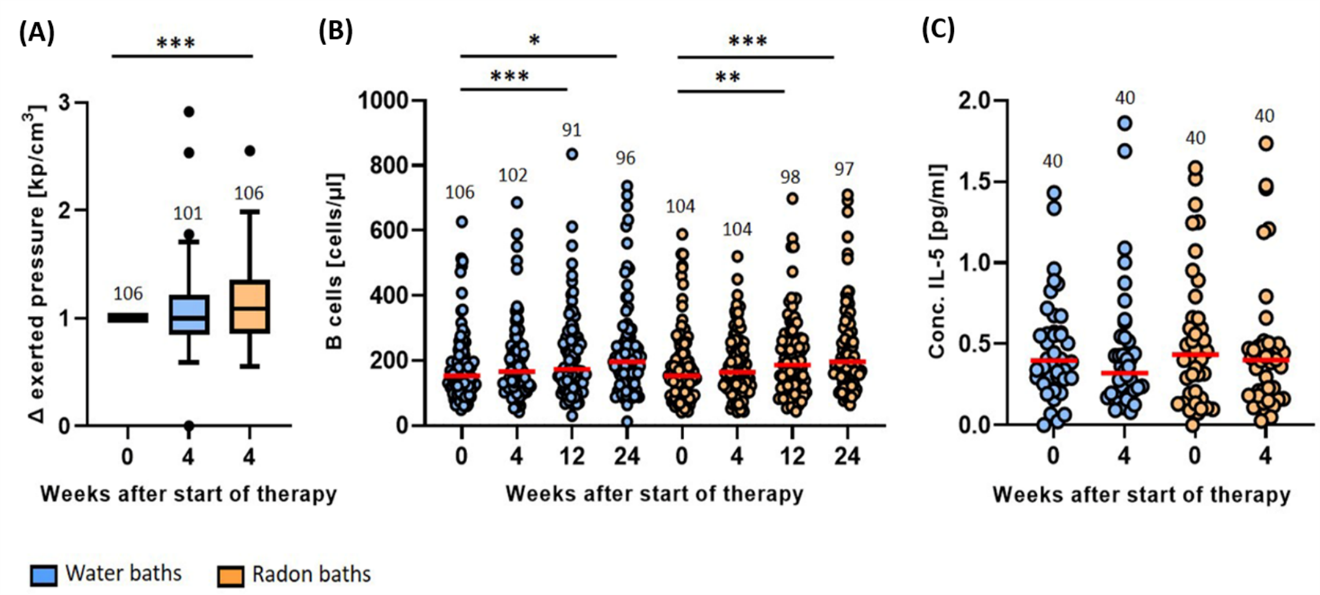


**Supplementary Figure 2.** **Comparison of the immune- and pain-related parameters to preclinical findings from a murine model.** The parameters assessed in the RAD-ON02 trial were compared to preclinical findings from a murine model on acute inflammatory arthritis after radon inhalation therapy. The pain levels were determined by pressure point algometry (kp/cm3) in week 0 and week 4 (a). The score achieved in week 4 was normalized to the initial pressure strength. The absolute count of B cells (cells/µl) was analyzed from peripheral blood at week 0, 4, 12 and 24 after each bath series by longitudinal immunophenotyping (b). The serum concentration of IL-5 (pg/ml) was determined by multiplex ELISA at week 0 and week 4 after both bath series (c). The Wilcoxon test was applied in (a) and (c). The paired t-test was applied in (b). The whisker in the boxplots were calculated by the Tukey method. Data points that were laying outside of these borders were plotted as black dots.
